# Exploring the Effects of Plant‐Based Ingredients and Phytochemicals on the Formation of Advanced Glycation End Products in Bakery Products: A Systematic Review

**DOI:** 10.1002/fsn3.70534

**Published:** 2025-06-30

**Authors:** Busra Turan‐Demirci, Buket Gonen‐Colak, Zehra Buyuktuncer

**Affiliations:** ^1^ Department of Nutrition and Dietetics, Faculty of Health Sciences Hacettepe University Ankara Turkey; ^2^ Department of Nutrition and Dietetics, Faculty of Health Sciences Cankiri Karatekin University Cankiri Turkey; ^3^ Department of Nutrition and Dietetics, Faculty of Health Sciences Bandirma Onyedi Eylül University Balıkesir Turkey

**Keywords:** advanced glycation end products, anti‐glycation activity, bakery products, Maillard reaction, phytochemicals, plant‐based ingredients

## Abstract

Bakery products are one of the most commonly consumed food groups in many countries. Advanced glycation end products (AGEs) occur during the baking process of bakery products, which are known to have adverse health effects. This systematic review assessed the potential of plant‐based ingredients and phytochemicals in reducing the formation of AGEs in bakery products. Following PRISMA guidelines, a search of PubMed, Web of Science, and Scopus on 4 July 2024 identified 1227 articles, and after applying the inclusion criteria, 26 studies were selected and reviewed in detail. The review found that plant‐based ingredients and phytochemicals can effectively inhibit the formation of various AGEs and precursors, with an inhibition rate ranging from < 10.00% to over 90.00%. All individual phytochemicals investigated for their anti‐glycation activity were polyphenols, and similarly, all plant‐based ingredients were the natural sources of polyphenols. However, determining the effective mechanisms to prevent AGE formation in the complex food matrices remains challenging. The mechanisms include scavenging free radicals, chelating metal ions, and inhibiting carbonyl formation. This systematic review highlights the potential of plant‐based interventions to mitigate AGE formation in bakery products. The insights gained from this study are expected to provide comprehensive guidance on the reformulation of bakery products to reduce AGE content, thereby supporting the prevention of AGE‐related diseases and contributing to the improvement of public health.

## Graphical abstract

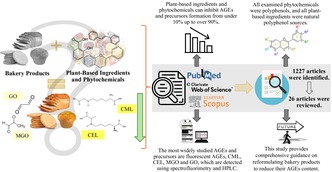

## 1 Introduction

**FIGURE 1:**
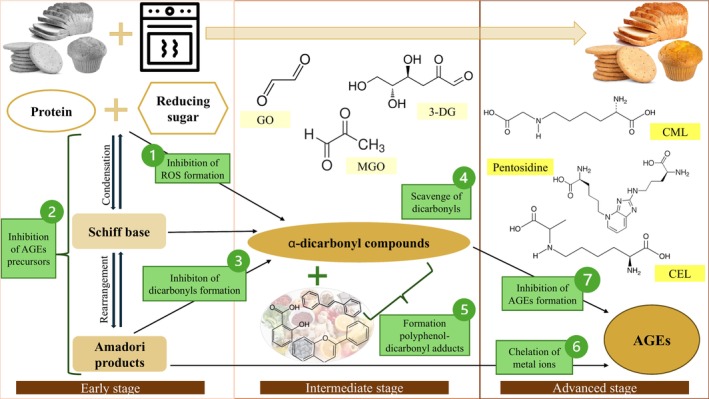
Formation of common AGEs in bakery products and prevention mechanisms.

Advanced glycation end products (AGEs) are complex compounds formed during the final stages of the Maillard reaction, a non‐enzymatic glycation that occurs when foods containing proteins and reducing sugars are exposed to heat. This process occurs between the amino groups of proteins and the carbonyl groups of reducing sugars (Kathuria et al. 2023) (Figure 1). Structural rearrangement of an unstable compound called the Schiff base, formed in the early step of the glycation process, results in relatively more stable Amadori products. In the intermediate stage, Amadori products undergo progressive dehydration and degradation to produce α‐dicarbonyl compounds such as glyoxal (GO), methylglyoxal (MGO), and 3‐deoxyglucosone (3‐DG) (Han et al. 2024). These highly reactive intermediates are important precursors of AGEs. In the advanced stages of glycation, they react with arginine and lysine residues in proteins to form highly stable, irreversible AGEs (Li, Zhuang, et al. 2023). A wide range of AGEs are found in food systems, including N‐ε‐carboxymethyllysine (CML), N‐ε‐carboxyethyllysine (CEL), pyrraline, and pentosidine. AGEs can be classified according to their molecular weight, fluorescence, and cross‐linking properties. CML, CEL, and free fluorescent AGEs are the most commonly used outcomes in AGEs inhibition studies in food systems (Melini et al. 2024).

The accumulation of AGEs in the body due to high dietary intake has been implicated in the development of several diseases, including obesity, diabetes and diabetic complications, cardiovascular disease, Alzheimer's disease, and cancer (Li, Zhuang, et al. 2023). Although the precise mechanisms underlying these adverse health effects of AGEs are not fully understood, it is hypothesized that AGEs contribute to disease progression by interacting with the receptor for AGEs (RAGE), altering intracellular signaling and gene expression, and promoting the release of pro‐inflammatory molecules and the generation of reactive oxygen species (ROS) (Takeuchi et al. 2022). These biochemical changes trigger chronic inflammation and increased oxidative stress, ultimately resulting in cellular damage, tissue dysfunction, and non‐communicable diseases (Garay‐Sevilla et al. 2020). Due to the negative effects of AGEs, the development of strategies to minimize the formation of AGEs in foods has become increasingly important to reduce dietary AGE intake (Li, Peng, et al. 2023).

Food processing methods involving dry heat significantly promote the formation of AGEs, especially in lipid‐ and protein‐dense food matrices. Bakery products are highly susceptible to the formation of AGEs during baking due to the presence of ingredients that are prone to the Maillard reaction, such as fat, sugar, and protein‐rich ingredients (Xu et al. 2024). Bread, cookies, and cakes have been identified as the primary bakery products associated with elevated AGEs levels (Boz 2023). Reports indicate that the annual per capita consumption of bread and bakery products ranges from 52 to 124 kg across various countries, and a 40.4% increase is projected by 2025 compared to 2021 (Mickiewicz and Britchenko 2022). This high consumption of bakery products significantly affects the overall intake of AGEs. Various strategies have recently been investigated to reduce AGE levels in bakery products. These include optimizing product formulation, improving cooling methods, and baking conditions such as baking temperature and time, using different baking equipment and technologies (Xu et al. 2023). The addition of functional phytochemicals that inhibit glycation is the most commonly used strategy to control AGE formation in bakery products.

Phytochemicals are secondary metabolites that occur naturally in fruits, vegetables, grains, and other plants and are typically responsible for organoleptic properties such as color, smell flavor, and texture. These biologically active constituents are reported to promote health and disease prevention through their antioxidant, antiinflammatory, antimicrobial, antimutagenic, and anticarcinogenic properties (Goswami et al. 2024). Thousands of phytochemicals have been identified, including carotenoids, polyphenols, phytosterols, specific polysaccharides, glucosinolates, terpenes, and saponins, which are classified according to their chemical structure and function. However, the precise categorization of phytochemicals remains a challenge due to their diverse forms and structures. The health benefits of extracts from some plants, known as medicinal plants, are also largely attributed to the phytochemicals present in their composition. In addition to their well‐known properties, recent studies have suggested that plant extracts and phytochemicals may also have anti‐glycation activity. This anti‐glycation activity is based on mechanisms such as scavenging free radicals and reducing the formation of reactive carbonyl compounds to alleviate oxidative stress, chelating metal ions as AGE formation is associated with the presence of transition metal ions, inhibiting carbonyl or dicarbonyl binding to proteins, and preventing the formation of late‐stage Amadori products (Wu et al. 2011) (Figure 1).

As bakery products are a staple food consumed daily by people of all ages in many countries, systematically reviewing the strategies for preventing AGE formation in these products and providing an overview of emerging approaches is critical for the protection of public health. The formation, detection, and various inhibition mechanisms of AGEs in bakery products have been investigated (Boz 2023; Xu et al. 2023) as well as the reduction of Maillard reaction products, including various compounds such as acrylamide, by phenolic compounds (Melini et al. 2024) and the overall inhibitory effect of phenolic compounds and plant extracts on the formation of AGEs (Khan et al. 2020). However, to the best of our knowledge, there is no systematic review in the existing literature that specifically evaluates the effects of directly adding phytochemicals and plant‐based ingredients to actual bakery products on AGE formation. The aim of this systematic review is to provide a comprehensive evaluation of the effectiveness of various phytochemicals and plant‐based ingredients in reducing levels of AGEs in real bakery product matrices and to address the existing knowledge gap in this field. This study is expected to provide insights into the reformulation of bakery products with reduced AGE content, thereby supporting the improvement of public health.

## 2 Methods

### 2.1 Search Strategy Protocol

This systematic review was conducted in accordance with the preferred reporting items for systematic reviews and meta‐analyses (PRISMA) guidelines (Supporting Information S1) (Page et al. 2021), independently by two researchers. First, the optimal keywords and synonyms were selected using MeSH terms (PubMed) and EMTREE (Scopus) and by examining keywords in relevant articles and reviews (Supporting Information S2). The literature search was conducted on 04 July 2024 in several international databases including PubMed, Web of Science, and Scopus. No date restrictions were applied. The search queries using the selected keywords in the specified databases are described in detail in Supporting Information S3. The web application “Rayyan” was used for the initial selection of studies after importing all the literature search data (Ouzzani et al. 2016). Simultaneously, a manual search of the references of the extracted articles was performed to ensure that no relevant studies were missed.

### 2.2 Inclusion and Exclusion Criteria

The screened articles were independently reviewed and assessed against the established research criteria. Each author reviewed all sections of the articles to determine which met the inclusion criteria. Any disagreements were resolved with the involvement of the third author. The inclusion criteria for this review are as follows: (1) full‐text articles published in English; (2) original research papers investigating the inhibition of AGEs (as concentration or percentage) by plant‐based ingredients or individual phytochemicals; and (3) studies focusing exclusively on bakery product samples. The following types of publications were excluded: book chapters, review articles, theses, clinical trial studies, and experimental studies investigating the anti‐glycation activity of plants or phytochemicals in different food groups or in vitro matrices other than bakery products.

### 2.3 Outcome Measures

The fluorescent AGEs, CML and CEL were selected as primary outcomes to investigate the inhibition of AGEs by plant‐based ingredients and phytochemicals. Secondary outcomes included other AGEs such as methylglyoxal‐lysine dimer (MOLD) and glyoxal‐lysine dimer (GOLD) as well as α‐dicarbonyl compounds such as GO, MGO, and 3‐DG, where data were available. Studies were selected based on their primary outcome measures.

### 2.4 Data Extraction

Data extraction included the type of bakery product, baking conditions (baking time and temperature), type of inhibitors, addition levels of inhibitors, inhibition rates (%) of selected outcomes, and methods of detection. The evaluation of included studies and the inhibition rates of AGEs in bakery products by different plant‐based ingredients and phytochemicals are presented in Table 1.

**TABLE 1:** Assessment of selected studies (*n* = 26) and inhibition rates of AGEs in bakery products by various plant‐based ingredients and phytochemicals.

References	Bakery product	Baking conditions	Inhibitors	Addition levels	Inhibition rates (%)					Studies outcome	Analytical methods
					Fluorescent AGEs	CML	CEL	Other AGEs	Dicarbonyl compounds		
Xu et al. (2024)	Bread	200°C for 10 min	Purified betacyanin‐rich extract (Dragon fruit peel, *Hylocereus polyrhizus* )	0.25%, 0.50%, 0.75%, 1.00%, and 2.00% w/w flour	Dose‐dependent reduction in both the crust and crumb					The formation of AGEs during baking was dose‐dependently suppressed by the incorporation of betacyanins into bread	Spectrofluorimetry
Zhou et al. (2023)	Bread	Bread machine	Spinach microgreen	80.00 g/bread	29.32 ± 4.43 (upper crust) 34.90 ± 1.76 (bottom crust)					The addition of spinach microgreen exhibited antiglycation activity on the bread crust	Spectrofluorimetry
				120.00 g/bread	57.18 ± 8.17 (upper crust) and 43.11 ± 5.15 (bottom crust)						
Liu et al. (2021)	Bread	170°C for 15 min	Alkylresorcinols	0.03% w/w flour		21.70				The addition of alkylresorcinols can have a significant dose‐dependent inhibitory effect on the formation of CML	LC–MS/MS
				0.10% w/w flour		35.11					
				0.30% w/w flour		42.18					
Teng, Li, et al. (2018)	Bread crust	Bread maker	Naringenin	0.25% (w/w)	11.79	19.67				The formation of CML and fluorescent AGEs was significantly inhibited by increasing the level of naringenin in bread crust	Spectrofluorimetry, ELISA
				0.50% (w/w)	~25.00	~40.00^b^					
				1.00% (w/w)	35.19	54.27					
Lin et al. (2018)	Steamed bread	10 min steaming	Quercetin	0.05%	~30.00^b^ (bread crumb)					Inclusion of quercetin at concentrations of 0.05%, 0.10% and 0.20% progressively increased the inhibition of fluorescent AGEs formation in steamed bread crumb and skin	Spectrofluorimetry
				0.10%	~50.00^b^ (bread crumb)						
				0.20%	58.60 (bread crumb)						
Lin and Zhou (2018)	Bread	200°C for 8 min	Quercetin	0.05%	~15.00–35.00^b^					The reduction of AGEs in both the crumb and crust of quercetin‐fortified bread increased linearly with the quercetin concentration, demonstrating a dose‐dependent effect	Spectrofluorimetry
				0.10%	~35.00–45.00^b^						
				0.20%	Above 55.00						
Mildner‐Szkudlarz et al. (2017)	Bread	210°C for 20 min	(+)‐Catechin	0.10 g/100 g of flour		Crust: 87.56^a^ Crumb: 78.44				The inhibition rates of CML formation ranged from 0.01% to 87.56% for bread crust and from 6.85% to 78.44% for bread crumb. Ferulic acid at higher concentrations (1.00%) and other phenolic acids at lower concentrations (0.10%) significantly inhibited CML formation in a dose‐dependent (but not linear) manner	HPLC
			Quercetin			Crust: 85.09^a^ Crumb: 69.72					
			Gallic acid			Crust: 56.29^a^ Crumb: 31.74					
			Caffeic acid			Crust: 81.07^a^ Crumb: 52.42					
			Ferulic acid	1.00 g/100 g of flour		Crust: 71.88^a^ Crumb: 53.15					
Peng et al. (2010)	Bread crust	Bread maker	Grape seed extract	0.30 g/500 g bread		Above 10.00^b^				Addition of grape seed extract dose‐dependently reduced CML content in bread crust	HPLC
				0.60 g/500 g bread		Over 30.00					
				1.00 g/500 g bread		Over 50.00					
Zhang et al. (2024)	Cookies	190°C for 15 min	Hesperetin	0.01% w/w	−10.97^a^					Hesperetin effectively inhibited the formation of fluorescent AGEs by scavenging MGO and forming two distinct hesperetin‐MGO adducts	Spectrofluorimetry
				0.05% w/w	51.17^a^						
				0.10% w/w	65.19^a^						
				0.30% w/w	80.77^a^						
				0.50%, w/w	83.47^a^						
Hsiao et al. (2024)	Cookies	200°C for 3 min	Blackcurrant extract	%5.00		49.18	46.18	MG‐H1: 31.73 G‐H1: 11.56 MOLD: 65.09 GOLD: 52.49	MGO: 60.33 GO: 51.18 3‐DG:46.83 3‐DGal: 53.00 Glucosone:26.72 DA: 17.07 (Diacetyl)	The addition of blackcurrant extract to the cookies significantly inhibited the formation of various AGEs, and α‐dicarbonyl compounds	UHPLC
Chumroenvidhayakul et al. (2023)	Cookies	170°C for 13 min	Dragon fruit peel powder ( *Hylocereus undatus* )	5.00% w/w	36.90				MGO: 52.10	The addition of dragon fruit peel powder resulted in a significant reduction in MGO and fluorescent AGEs, with the greatest reduction observed at the 5.00% concentration, followed by the 2.00% and 1.00% concentrations	Spectrofluorimetry, HPLC
Hu et al. (2022)	Cookie	170°C for 12 min	Catechins	0.30%, 0.70%, 1.00%, 2.00%, 3.00%, 4.00% and 5.00% w/w		Free CML and CEL: 31.89–84.19 Protein‐bound CML: 19.88–26.71	Free CML and CEL: 31.89–84.19 Protein‐bound CEL: 15.32–30.64		MGO, GO and 3‐DG: Effectively inhibited	Catechins have a strong inhibitory effect on the formation of CML, CEL and α‐dicarbonyl compounds. This effect is dose‐dependent for CML and CEL, but less clear for other compounds	HPLC
			Curcumin			Free CML: 46.62 Protein‐bound CML: 11.39%–27.70%	Free CEL: 15.35–25.34 Protein‐bound CEL: NI (promote in high concentration)		MGO, GO and 3‐DG: Not inhibited		
Mildner‐Szkudlarz et al. (2022)	Biscuit	180°C for 12 min	Grape by‐product	10.00 g/100 g flour		89.00				It is possible to produce biscuits with low levels of CML by adding grape by‐products to a basic wheat recipe	HPLC
Chen, Tan, et al. (2022)	Biscuit	170°C for 15 min	Lotus seedpod oligomeric procyanidins	0.50 mg/g biscuits	~20.00^b^	17.67 ± 4.62				The reduction in fluorescent AGEs and CML levels increased with higher concentrations of lotus seedpod oligomeric procyanidins	Spectrofluorimetry, HPLC
				4.00 mg/g biscuits	39.79 ± 0.34	93.14 ± 2.66					
Wu et al. (2022)	Cookie	180°C for 15 min	Lotus seedpod oligomeric procyanidins	0.05%	7.80	~30.00^b^				The levels of fluorescent AGEs and CML decreased as the concentration of lotus seedpod oligomeric procyanidins increased	Spectrofluorimetry, HPLC
				0.10%	22.35	~50.00^b^					
				0.20%	47.62	~70.00^b^					
				0.40%	52.12	~90.00^b^					
Chen, Lin, et al. (2022)	Cookie	200°C for 16 min	Dried extract of rooibos ( *Aspalathus linearis* )	0.50%, 1.00% (w/w)		~45.00–70.00^b^	~45.00–70.00^b^	MG‐H1: ~50.00–70.00^b^ G‐H1: ~40.00–60.00^b^ MOLD: ~50.00–75.00^b^ GOLD: ~60.00–80.00^b^	MGO: ~60.00–75.00^b^ GO: ~55.00–60.00^b^ 3‐DG: ~60.00–70.00^b^ 3‐DGal:~40.00–55.00^b^ Glucosone: ~10.00–40.00	Rooibos addition led to a significant, dose‐dependent reduction in both α‐dicarbonyls and AGEs	LC–MS/MS
Troise et al. (2020)	Cookie	180°C for 13 min	Spray‐dried olive mill wastewater	0.05%, 0.10% and 0.20%		20.00	Reduced significantly		MGO: Reduced significantly GO: 80.00	Olive oil mill wastewater polyphenol powders significantly inhibited the formation of CML (only %0.20), CEL and dicarbonyls in cookies at three different concentrations	LC–MS/MS
Gao et al. (2020)	Cookie	170°C/150°C (upper/lower) for 5 min	Apple flower powder	1.00% w/w	11.18				MGO: 11.82	The apple flower powder significantly inhibited the formation of methylglyoxal and fluorescent AGEs	Spectrofluorimetry, HPLC
				2.50% w/w	22.05				MGO: 57.33		
				5.00% w/w	44.77				MGO: 60.36		
Ou et al. (2018)	Cookie	150°C, 170°C and 190°C for 10 min	Resveratrol	0.02% w/w	~20.00–30.00^b^				MGO: −3.7 to 53.3^a^ GO: 5.7–14.3^a^	Rosmarinic acid showed the most significant inhibitory effect on fluorescent AGEs, followed by resveratrol and epicatechin, with higher polyphenol concentrations enhancing this inhibition Epicatechin and resveratrol significantly reduced GO and MGO in cookies at all temperatures. In comparison, rosmarinic acid did not affect GO and only decreased MGO at higher concentrations	Spectrofluorimetry, HPLC
				0.20% w/w	~40.00–60.00^b^				MGO: 32.5–52.1^a^ GO: 17.1–28.0^a^		
			Epicatechin	0.02% w/w	~20.00–30.00^b^				MGO: 16.20–38.10^a^ GO: 0.60–11.10^a^		
				0.20% w/w	~30.00–40.00^b^				MGO: 25.50–64.60^a^ GO: 16.00–41.70^a^		
			Rosmarinic acid	0.02% w/w	~30.00–40.00^b^				MGO: −10.10 to −2.30^a^ GO: −4.50 to 1.10^a^		
				0.20% w/w	~40.00–60.00^b^				MGO: 24.20–35.80^a^ GO: −1.70 to 9.10^a^		
Huang et al. (2018)	Biscuit	190°C for 8 min	Maize bran feruloylated oligosaccharides	10.00 mg/g flour	80.00				MGO: 67.00 GO: 54.00 3‐DG: 46.00	The addition of feruloylated oligosaccharides significantly reduced fluorescent AGEs, 3‐DG, GO and MGO in a dose‐dependent manner However, increasing the FO concentration above 20.00 mg/g did not further reduce fluorescent AGEs and only gradually reduced dicarbonyl levels	Spectrofluorimetry, HPLC
				20.00 mg/g flour	90.00				NA		
Teng, Liu, et al. (2018)	Cookie	180°C for 20 min	Naringenin	0.25% (w/w)	18.30	Not inhibited				Dihydromyricetin showed the most potent effect in reducing levels of CML and fluorescent AGEs	Spectrofluorimetry, ELISA
			Naringin		Not inhibited	Not inhibited					
			Hesperetin		31.40	Not inhibited					
			Dihydromyricetin		47.00	Significantly inhibited					
Navarro and Morales (2017)	Biscuit	180°C for 20 min	Hydroxytyrosol	0.25%, 0.50% and 0.75% (w/w)	28.80	Not inhibited	23.30	Pentosidine: 22.80–34.30	MGO: Not inhibited GO: Not inhibited 3‐DG: 37.90–41.60	The biscuits formulated with hydroxytyrosol significantly reduced the formation of fluorescent AGEs, pentosidine, CEL and 3‐DG. Olive leaf extract showed comparable antiglycation effects on pentosidine and a greater effect on CEL at low concentrations	Spectrofluorimetry, HPLC, LC‐MS/MS
			Olive leaf extract	0.01% and 0.05% w/w	Below 10.00	Not inhibited	42.16	Pentosidine: 37.30	MGO: Not inhibited GO: Not inhibited		
			Quercetin	0.25% w/w	64.90	Not inhibited	21.50	Pentosidine: 59.50	MGO: Not inhibited GO: Not inhibited		
			Gallic acid	0.25% w/w	Below 10.00	Not inhibited	49.19	Pentosidine: 52.20	MGO: Not inhibited GO: Not inhibited		
Zhang et al. (2014)	Cookie	200°C for 10 min	Naringenin	0.25% (w/w)	~20.00^b^				MGO: Not inhibited GO: ~20.00^b^	Compared with other tested polyphenols, quercetin is suggested to be the most promising functional cookie additive given strong inhibition against AGEs	Spectrofluorimetry, HPLC
			Quercetin		Above 80.00				MGO: Not inhibited GO: ~50.00^b^		
			Epicatechin		~15.00^b^				MGO: Not inhibited GO: ~40.00^b^		
			Chlorogenic acid		Not inhibited				MGO: Not inhibited GO: ~10.00^b^		
			Rosmarinic acid		~15.00^b^				MGO: Not inhibited GO: ~20.00^b^		
Przygodzka and Zieliński (2016)	Rye‐buckwheat ginger cakes	180°C for 18 min	Rutin	50.00 mg		32.43^a^				The addition of rutin significantly reduced CML formation in cakes made with light buckwheat flour and rye flour, while this effect was not observed in cakes made with flour from roasted buckwheat dehulled grains and rye flour	HPLC
				100.00 mg		34.93^a^					
Mildner‐Szkudlarz et al. (2015)	Muffin	180°C for 20 min	Grape by‐product	%20.00		16.20–93.30^a^				The addition of GP to muffins of different recipes resulted in a significant reduction in CML levels, and in some recipes enriched with GP, CML levels remained below the limits of detection	HPLC
Srey et al. (2010)	Sponge cake	190°C for 30 min	Ferulic acid	Molar ratio of lysine:inhibitör (1:1, 1:5)		Significantly inhibited	Significantly inhibited			Ferulic acid effectively reduced both CML and CEL levels while rutin did not inhibit CML and CEL formation	UHPLC
			Rutin			Not inhibited	Not inhibited				

^a^ The percentage of inhibition was calculated based on the concentration reported in the article.

^b^ The percentage of inhibition was estimated from the graphical values as it was not given numerically in the article.

## 3 Results

**FIGURE 2:**
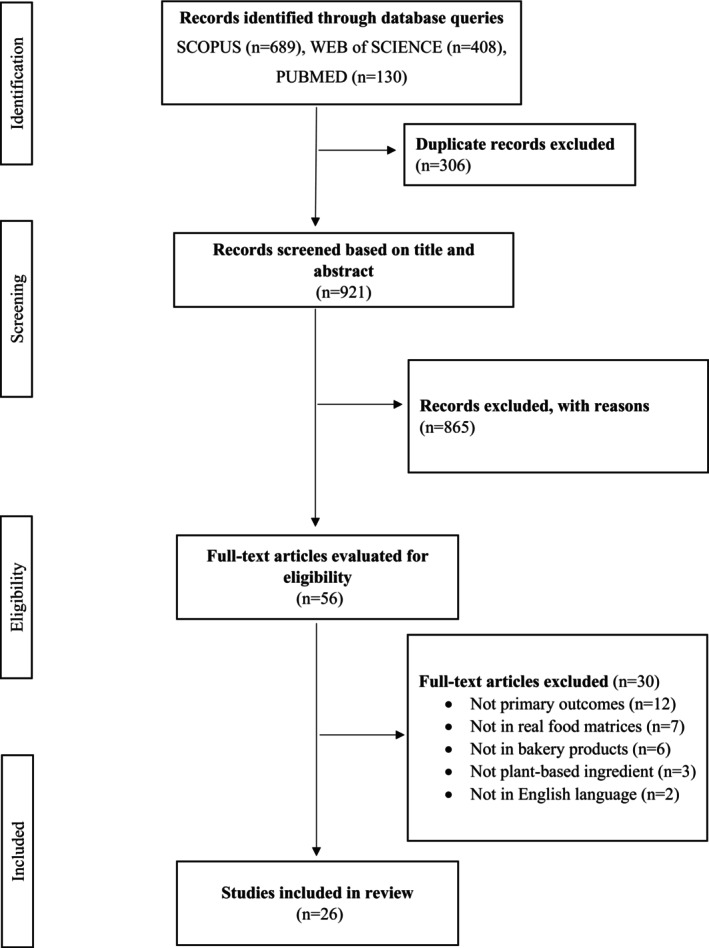
The PRISMA flowchart.

The PRISMA flowchart in Figure 2 illustrates that 1227 articles were identified by searching three different databases. Using the Rayyan, 306 duplicate records were removed, and the remaining 921 records were screened based on titles and abstracts to exclude those clearly irrelevant. After a comprehensive evaluation of 56 articles, 26 articles that met the specified inclusion criteria were included in this systematic review. These articles, published between 2010 and 2024, are summarized in Table 1.

The studies focusing on the bakery products included examples of bread (8 articles), cookies (11 articles), biscuits (4 articles), and cakes (3 articles). These studies investigated the anti‐glycation activity of both plant‐based ingredients and individual phytochemicals. The plant‐based ingredients included dragon fruit extract (2 articles), blackcurrant extract (1 article), spinach microgreen (1 article), grape seed extract (1 article), grape by‐product (2 articles), olive leaf extract (1 article), spray‐dried olive mill wastewater (1 article), apple flower powders (1 article), dried extract of rooibos (1 article), and maize bran feruloylated oligosaccharides (1 article). The individual phytochemicals included hydroxytyrosol (1 article), alkylresorcinols (1 article), naringenin (3 articles), quercetin (5 articles), catechins (4 articles), gallic acid (2 articles), caffeic acid (1 article), ferulic acid (2 articles), hesperetin (1 article), curcumin (1 article), resveratrol (1 article), rosmarinic acid (2 articles) (1 article), naringin (1 article), dihydromyricetin (1 article), chlorogenic acid (1 article), rutin (2 articles), and lotus seedpod oligomeric procyanidins (2 articles). Concentrations of these plant‐based ingredients and phytochemicals varied widely, ranging from 0.01% to 5.00% (weight/weight, w/w). Similarly, the cooking conditions involved temperatures ranging from 150°C to 210°C, with baking times of between 3 and 30 min. The inhibition rates of AGEs by these components ranged from < 10.00% to more than 90.00%, as detailed in Table 1.

**FIGURE 3:**
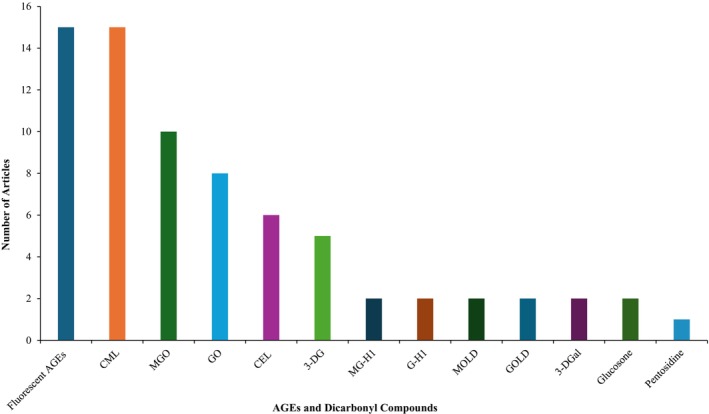
Number of articles of AGEs and dicarbonyl compounds studied for AGE reduction studies in bakery products.

In the articles selected according to the primary outcomes, the analyzed AGEs included fluorescent AGEs (15 articles), CML (15 articles), CEL (6 articles), MG‐H1 (2 articles), G‐H1 (2 articles), MOLD (2 articles), GOLD (2 articles), and pentosidine (1 article). Dicarbonyl compounds analyzed were MGO (10 articles), GO (8 articles), 3‐DG (5 articles), 3‐DGal (2 articles), and glucosone (2 articles) (Figure 3).

**FIGURE 4:**
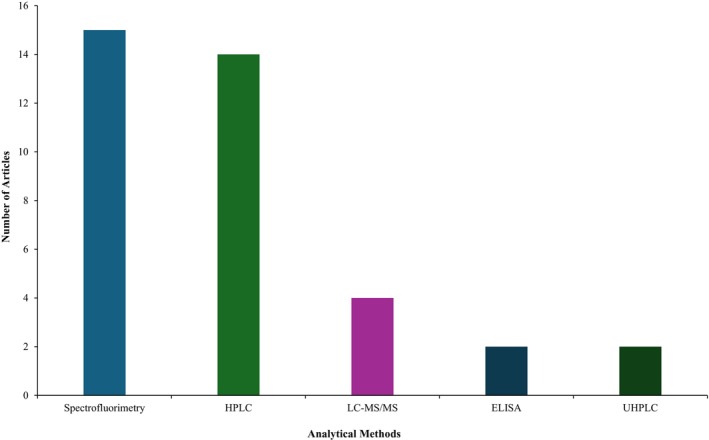
Number of articles on analytical techniques for AGE detection.

A variety of techniques are used to identify and measure the AGEs and dicarbonyl compounds. The methods used in the selected studies included spectrofluorimetry, high‐performance liquid chromatography (HPLC), liquid chromatography‐ tandem mass spectrometry (LC–MS/MS), ultra‐high‐performance liquid chromatography (UHPLC), and enzyme‐linked immunosorbent assay (ELISA), as shown in Figure 4. Spectrofluorimetry was the most commonly used method to detect the fluorescent AGEs, and HPLC was the second most commonly used technique, mainly to identify the other types of AGEs and dicarbonyl compounds.

## 4 Discussion

To our knowledge, this is the first study that specifically examined the ability of plant‐based ingredients and individual phytochemicals, which were added to real bakery food matrices to reduce various AGEs and their precursors, α‐dicarbonyl compounds. Research that examines the potential of natural compounds in the prevention of AGE formation is relatively recent, and there is a growing interest in screening for natural anti‐glycation agents (Song et al. 2021; Wu et al. 2011). The novelty of this field is highlighted by the inclusion of studies dating back to 2010 in our review.

Although the anti‐glycation activity of phytochemicals and plant extracts has been studied more frequently using in vitro models (Khan et al. 2020; Song et al. 2021), it is challenging to identify their potential in a real food system. This challenge is especially pronounced in complex bakery products such as bread, biscuits, and cakes, where a diverse range of ingredients complicates the identification of anti‐glycation mechanisms (Kocadağlı et al. 2016). In addition, the differences in the weight and thickness of the samples, cooking times and temperatures, and the use of non‐standard cooking equipment further complicate the interpretation of the results (Melini et al. 2024). Of the included studies, almost all were conducted at different baking temperatures, times, and conditions. For this reason, this study evaluated the role of plant‐based ingredients and phytochemicals in the prevention of AGE formation based on the inhibition percentages rather than the concentrations. Plant‐based ingredients and phytochemicals examined in the studies were found to successfully prevent the formation of some AGEs and dicarbonyls in bread, cookies, and cakes (Table 1). The wide variation in the reported AGE inhibition rates can be mainly attributed to factors such as interactions with complex food matrices and the thermal stability of inhibitory compounds during the cooking process (Kocadağlı et al. 2016).

### 4.1 Using Phytochemicals in the Prevention of AGE Formation in Bakery Products

All individual phytochemicals investigated in the studies for their anti‐glycation activity belong to the polyphenol group, which includes flavonoids (quercetin, hesperetin, naringenin, naringin, dihydromyricetin, catechins, rutin, and lotus seedpod oligomeric procyanidins), phenolic acids (ferulic acid, gallic acid, caffeic acid, rosmarinic acid, and chlorogenic acid), stilbenes (resveratrol), and other polyphenols (hydroxytyrosol, curcumin, and alkylresorcinols) (Table 1). Although the search process used comprehensive keywords to include polyphenols, non‐polyphenol phytochemicals, and all plant‐based compounds, no studies on non‐polyphenol phytochemicals were identified after screening the results according to the inclusion criteria. Limited research into other classes of phytochemicals, such as alkaloids, terpenoids, and organosulfur compounds, suggests a gap in the literature. Polyphenols are the most extensively studied natural compounds for their role as anti‐glycation agents in foods (Shi et al. 2024; Khan et al. 2020). Their anti‐glycation activity is primarily attributed to their antioxidant capabilities and their ability to scavenge dicarbonyl compounds. These antioxidants inhibit AGE formation by chelating metal ions, scavenging free radicals, and reducing oxidative stress. They also scavenge carbonyl compounds formed during the intermediate stages of glycation and thus contribute to their overall inhibitory effect (Wu et al. 2011).

#### 4.1.1 Flavonoids

The anti‐glycation activity of flavonoids such as quercetin, naringenin, and catechins on various bakery products has been studied several times (Table 1). The addition of 0.10%–0.20% quercetin to breads reduced both fluorescent AGEs and CML levels by over 50.00% compared to control samples (Lin et al. 2018; Lin and Zhou 2018; Mildner‐Szkudlarz et al. 2017). Similarly, adding 0.25% quercetin to biscuits significantly reduced fluorescent AGEs, CEL, and pentosidine by 64.90%, 21.50%, and 59.50%, respectively, but did not significantly affect CML or dicarbonyl compounds such as MGO and GO (Navarro and Morales 2017). Likewise, quercetin added to cookies at the same concentration was suggested as the most promising functional additive, as it provided a significant inhibition of fluorescent AGEs and GO, compared to naringenin, epicatechin, chlorogenic acid, and rosmarinic acid (Zhang et al. 2014). Quercetin, a prominent flavonol found in foods such as apples, onions, and berries, exhibits powerful antioxidant activity. The anti‐glycation mechanism of quercetin is proposed to primarily involve the trapping of MGOs and the subsequent formation of mono‐ and di‐MGO adducts (Li et al. 2014). Another flavonol studied for its anti‐glycation activity in bakery products is rutin (quercetin‐3‐rutinoside), a glycoside of quercetin, consisting of the quercetin attached to a rutinose sugar molecule (Ganeshpurkar and Saluja 2017). Adding 50.00–100.00 mg of rutin to rye‐light buckwheat flour ginger cakes reduced CML formation by 32.43%–34.93% (Przygodzka and Zieliński 2016). However, in another study with cakes, rutin did not inhibit the formation of CML and CEL (Srey et al. 2010). It has been reported that rutin exhibits significant inhibitory activity both in the early and intermediate stages of protein glycation, largely due to its potent antioxidant activity derived from the phenolic rings and free hydroxyl groups in its chemical structure (Wu and Yen 2005). However, the studies reporting its anti‐glycation activity were conducted in simple model systems, not in complex food matrices. In the case of bakery products, inconsistent results are explained by the difference in the type of bakery product, including different baking conditions or formulations that can influence the efficacy of rutin.

Naringenin and its glycoside form naringin are also the most extensively studied flavonoids for their potential in the prevention of AGE formation in bakery products. Naringenin is a flavanone found in abundance in citrus fruits and tomatoes. Teng, Li, et al. (2018) reported that the addition of naringenin at concentrations of 0.25%–1.00% reduced the levels of fluorescent AGEs and CML in bread crust by 11.79%–35.19% and 19.67%–54.27%, respectively. In cookies, the addition of 0.25% naringenin reduced fluorescent AGEs by 18.30% but had no significant effect on CML levels. Notably, naringin did not affect either CML or fluorescent AGE formation in the same study (Teng, Li, et al. 2018). This lack of effectiveness can be attributed to glycosylation, which reduces the antiradical capacity of the flavonoid by decreasing the number of free hydroxyl groups, disrupting the ortho‐hydroxyl structure, and obstructing the access of radical scavengers to the radical center (Cavia‐Saiz et al. 2010). Similarly, Zhang et al. (2014) found that naringenin at a concentration of 0.25% reduced fluorescent AGEs and GO levels in cookies by approximately 20.00% but did not significantly affect MGO levels. The lower anti‐glycation activity of naringenin compared to other flavonoids may be due to the absence of the C‐3′ hydroxyl group, as the efficacy of flavonoids in inhibiting protein glycation is closely linked to their structural characteristics (Wu and Yen 2005).

Hesperetin, which differs from naringenin in having an additional methoxy group, is another citrus‐derived flavanone, and two separate studies investigated its anti‐glycation activity in bakery products. One of these studies reported that the addition of hesperetin into cookies at the concentrations between 0.05% and 0.50% resulted in a dose‐dependent reduction in the formation of fluorescent AGE levels, ranging from 50.17% to 83.47% (Zhang et al. 2024). This reduction was explained by the scavenging of MGO and the formation of two different hesperetin‐MGO adducts. In the other study, the addition of 0.25% hesperetin resulted in a 31.40% reduction of fluorescent AGE levels; however, no significant change in CML levels was reported (Teng, Liu, et al. 2018). Dihydromyricetin, another flavonoid examined in this study, exhibited a greater reduction in fluorescent AGE levels (47.00%), particularly in CML levels, compared to naringenin, naringin, and hesperetin. The ability of polyphenols to inhibit AGE formation was positively correlated with their antioxidant activity, attributing this effect to the free radical scavenging properties of the phenolics. Although dihydromyricetin, the primary compound in dried stems and leaves of *Ampelopsis grossedentata*, is highly unstable under heat (only 36.10% remained after cooking at 180°C for 20 min), its significant anti‐glycation activity is likely to be enhanced by the bioactive process‐derived polyphenol degradation products (Debelo et al. 2020).

Catechins, a subclass of flavonoids known as flavanols, are present in various foods and plants including tea, apples, dates, cocoa, grapes, and berries. Their prominent examples are (+)‐catechin, (−)‐epicatechin, (+)‐gallocatechin, (−)‐epigallocatechin, and (−)‐epigallocatechin 3‐O‐gallate (Isemura 2019). Mildner‐Szkudlarz et al. (Mildner‐Szkudlarz et al. 2017) reported that adding (+)‐catechin to bread at a concentration of 0.10% reduced the CML levels by over 80.00%, but this inhibition rate decreased at higher concentrations of 0.50%, 1.00%, and 2.00%. The presence of catechins in the bread matrix can impact viscoelastic properties through possible interaction with components such as the gluten network, thereby affecting the migration and concentration of AGE precursors and indirectly reducing AGE formation (Goh et al. 2015). The addition of 0.30%–5.00% catechins to cookies reduced free CML and CEL levels by 31.89%–84.19% in a dose‐dependent manner. For the non‐free forms, catechins significantly reduced protein‐bound CML by 19.88%–26.71% at higher doses and protein‐bound CEL by 15.32%–30.64%, exhibiting a less pronounced dose dependence. Similar effects have been observed for α‐dicarbonyl compounds such as MGO, GO, and 3‐DG (Hu et al. 2022). Likewise, adding 0.02% and 0.20% epicatechin to cookies baked at three different temperatures reduced the levels of fluorescent AGEs, MGO, and GO by up to 40.00%, 64.60%, and 41.70%, respectively, with higher concentrations leading to greater inhibition rates at all temperatures (Ou et al. 2018). Zhang et al. (2014) also reported that a similar amount of epicatechin reduced fluorescent AGEs and GO in cookies by 15.00% and 40.00%, respectively, but did not inhibit MGO formation. In addition to strong antioxidant activities, catechins often possess hydroxyl groups at key positions that enable them to effectively scavenge reactive dicarbonyl species and inhibit the formation of AGEs (Sang et al. 2007). Two studies conducted by the same research group demonstrated that adding 0.05% to 0.40% of oligomeric procyanidins derived from lotus seedpods to cookies reduced fluorescent AGEs levels by 7.00% to 52.00% and CML levels by 17.00% to 93.00% (Chen, Tan, et al. 2022; Wu et al. 2022). Procyanidins consist of different proportions of (+)‐catechin, (−)‐epicatechin, and their derivatives, and their bioactive properties are affected by factors such as the degree of polymerization and the binding sites (Li et al. 2015). Similarly, the AGE inhibition properties of different flavonoids have been reported to vary, which is attributed to differences in the number and position of hydroxyl groups in their structures (Qin et al. 2021).

#### 4.1.2 Phenolic Acids

Studies investigating the effects of phenolic acids on AGEs formation in bakery products included gallic acid from hydroxybenzoic acids and ferulic, rosmarinic, caffeic, and chlorogenic acids from hydroxycinnamic acids (Table 1). Gallic acid is a widely distributed phenolic acid found in fruits such as pomegranate, grape, and hawthorn as well as in various herbal teas (Xiang et al. 2024). Navarro and Morales (2017) reported that adding 0.25% gallic acid to biscuits reduced CEL levels by 49.19% and pentosidine levels by 52.20% but did not affect the levels of CML or dicarbonyl compounds such as MGO and GO significantly. In breads, adding 0.10 g of gallic acid to 100 g of flour reduced CML levels in the crust by 56.29% and in the crumb by 31.74%. However, the higher concentrations of gallic acid exhibited lower anti‐glycation activity (Mildner‐Szkudlarz et al. 2017). Similarly, caffeic acid, another phenolic acid examined in the same study, demonstrated the highest efficiency at its lowest concentration, achieving an 81.07% reduction in CML levels in the bread crust and a 52.42% reduction in the crumb. It was suggested that certain phenolic compounds may exhibit lower anti‐glycation activity at higher concentrations due to their potential prooxidant activity at the initiation reactions (Gulcin 2020). Conversely, ferulic acid showed the highest anti‐glycation activity at a 10‐fold higher concentration (1.00 g/100 g flour) in bread samples, resulting in a reduction of CML levels by 71.88% in the crust and by 53.15% in the crumb (Mildner‐Szkudlarz et al. 2017). In another study focused on the anti‐glycation effects of ferulic acid, it was shown that ferulic acid can significantly inhibit the formation of both CML and CEL in cakes (Srey et al. 2010). Ferulic acid primarily inhibits AGE formation by reducing the production of precursors through its antioxidant activity, binding to amino groups, and preventing sugar autoxidation and the degradation of early Maillard reaction products (Silván et al. 2011). However, the results of these studies highlight the importance of carefully evaluating the dose–response relationship of each plant‐based ingredient and phytochemical in preventing glycation, particularly in bakery products (Mildner‐Szkudlarz et al. 2017).

Rosmarinic acid, an ester of 3,4‐dihydroxyphenyllactic acid and caffeic acid, is abundant in the Lamiaceae family, including rosemary, spearmint, and lemon balm (Petersen and Simmonds 2003). Adding rosmarinic acid to cookies at the concentrations of 0.02% and 0.20% reduced fluorescent AGE levels by approximately 30.00%–40.00% and 40.00%–60.00%, respectively, while higher concentrations also decreased MGO levels by 24.20%–35.80% (Ou et al. 2018). Similarly, Zhang et al. (2014) reported that adding 0.25% rosmarinic acid to cookies reduced fluorescent AGE levels by about 15.00% and decreased GO levels by approximately 20.00%. Chlorogenic acid, another hydroxycinnamic acid examined in the same study, did not reduce fluorescent AGE levels but resulted in a modest decrease in GO levels by 10.00%. Chlorogenic acid, a common phenolic compound found in foods such as coffee and tea, is known for its antioxidant properties (Naveed et al. 2018).

The diverse anti‐glycation effects of phenolic acids, despite their notable antioxidant properties, suggest that AGEs inhibition may involve mechanisms beyond their antioxidant activity. Other possible mechanisms are related to the intermediate reactions of the Maillard reaction, including the formation of less reactive adducts with carbonyl compounds, binding to key amino acid residues, the impacts on food matrix properties, the synergistic effects with other antioxidants, and the inhibition of enzymes involved in AGE formation (Dong, Zhang, et al. 2023; Anwar et al. 2021; Yeh et al. 2017).

#### 4.1.3 Stilbenes

Stilbenes, a small group of polyphenols, are derived primarily from trans‐resveratrol, with resveratrol (3,5,4′‐trans‐trihydroxystilbene) being a notable example of this family. Resveratrol, found in red grapes, berries, peanuts, and some vegetables, has been shown to have anti‐glycation properties in numerous in vitro studies (Bo et al. 2023). Ou et al. (2018) reported that adding 0.02% resveratrol to cookies reduced fluorescent AGE levels by approximately 20.00%–30.00%, while addition of 0.20% resveratrol reduced them by about 40.00%–60.00%. Additionally, resveratrol was shown to decrease MGO levels by 53.30% and GO levels by up to 28.00%. Resveratrol's potential to inhibit the formation of AGEs is likely due to its capacity to scavenge reactive oxygen species, form the covalent bonds with carbohydrates, and directly trap dicarbonyls to create dicarbonyl‐resveratrol adducts (Shen et al. 2017). Although there is only one study examining the effects of resveratrol in bakery products, it remains an important phytochemical with its anti‐glycation activity, as shown in the studies involving plant‐based ingredients, particularly those derived from grapes.

#### 4.1.4 Other Polyphenols

Other polyphenols, including alkylresorcinols, hydroxytyrosol, and curcumin, have been investigated for their potential to reduce AGE formation in bakery products (Table 1). The addition of alkylresorcinol to bread at concentrations of 0.03% and 0.30% reduced CML levels by 21.70% and 42.18%, respectively (Liu et al. 2021). Alkylresorcinols, primarily found in the outer layers of wheat, rye, and barley kernels, were used as biomarkers for whole grain intake. The inhibition of CML formation in bread by alkylresorcinols is likely attributed to their chemical structure and their capacity to reduce oxidative damage and reactive oxygen species (Zabolotneva et al. 2022).

Hydroxytyrosol, an essential component of oleuropein, is found in substantial quantities in olive oil and mill waste and is derived from the hydrolysis of oleuropein during olive maturation (Bertelli et al. 2020). In biscuits containing hydroxytyrosol at concentrations between 0.25% and 0.75%, the levels of fluorescent AGEs (28.80%), CEL (23.30%), pentosidine (22.80%–34.30%), and 3‐DG (37.90%–41.60%) were reduced, but CML levels remained statistically unchanged (Navarro and Morales 2017). The anti‐glycation mechanism of hydroxytyrosol is attributed to its chemical structure, which enables the effective trapping of reactive dicarbonyl species (Navarro and Morales 2016). Similarly, curcumin, the main curcuminoid in *Curcuma Longa* (Turmeric), has also exhibited anti‐glycation activity, mainly MGO‐trapping properties (Sun et al. 2016). The addition of curcumin to biscuits reduced free CML levels by up to 46.62%, free CEL by 15.35%–25.34%, and protein‐bound CML by 11.39%–27.70%; it did not significantly affect the formation of either protein‐bound CEL or α‐dicarbonyl compounds such as MGO, GO, and 3‐DG (Hu et al. 2022).

### 4.2 Using Plant‐Based Ingredients to Prevent AGE Formation in Bakery Products

Studies on anti‐glycation agents in bakery products have examined a range of sources, including plant extracts (grape seed, blackcurrant, dragon fruit peel, olive leaf, and rooibos), waste by‐products (grape and olive mill wastewater), plant materials (spinach microgreens), plant powders (dragon fruit peel and apple flower), and plant‐derived components (maize bran feruloylated oligosaccharides) (Table 1). Among these, grape‐derived ingredients are the most extensively studied, focusing on their application in a variety of matrices, including bread, biscuits, and cakes. Peng et al. (2010) reported that grape seed extract significantly reduced the level of CML in bread crust by more than 50.00% in a dose‐dependent manner. Similarly, the addition of 10.00% grape by‐product in cookies resulted in an 89.00% reduction in CML levels, while the addition of 20.00% grape by‐product in muffins resulted in an even greater reduction, up to 93.30% (Mildner‐Szkudlarz et al. 2022; Mildner‐Szkudlarz et al. 2015). The anti‐glycation activity of grape‐derived products is primarily attributed to grape polyphenols, including resveratrol, catechins, procyanidins, quercetin, and gallic acid (Coelho et al. 2023). In addition to the individual mechanisms related to each grape phenolic compound's activity, their combined synergistic effects are also significant in inhibiting AGE formation (Sri Harsha and Lavelli 2019). This synergistic effect may explain why grape‐derived ingredients have greater anti‐glycation activity compared to the effects of their individual phenolics in bakery products.

In a similar context, Hsiao et al. (2024) assessed the anti‐glycation activity of blueberry, strawberry, raspberry, blackberry, and blackcurrant in BSA‐glucose and BSA‐MGO models and found that blackcurrant had the highest anti‐glycation activity among the berries tested. Consequently, the addition of 5.00% blackcurrant extract, which showed the highest anti‐glycation activity, to cookies resulted in a 49.18% reduction in CML levels, a 46.18% reduction in CEL levels, and also reduction at the levels of other AGEs and α‐dicarbonyl compounds. These anti‐glycation effects are attributed to anthocyanins, such as the cyanidin and delphinidin glycosides found in blackcurrant, which can act as potent carbonyl scavengers by forming mono‐ and di‐MGO adducts (Hsiao et al. 2024).

The anti‐glycation activity of dragon fruit peel, both in extract and powder forms, were examined in various bakery products. Adding 0.25%, 0.50%, 0.75%, 1.00%, and 2.00% (w/w) of a purified betacyanin‐rich extract obtained from *Hylocereus polyrhizus* peel to bread resulted in a dose‐dependent suppression of AGE formation (Xu et al. 2024). Similarly, the addition of dragon fruit ( *Hylocereus undatus* ) peel powder to cookies resulted in a significant reduction in fluorescent AGEs (%36.90) and MGO (%52.10), with the greatest reduction observed at the 5% concentration, followed by the 2.00% and 1.00% concentrations (Chumroenvidhayakul et al. 2023). The inhibition of AGE formation by the dragon fruit‐derived ingredients is due to the biological activity of its components including phenolic acids and betacyanins. These bioactive components have ability to scavenge free radicals, bind metal ions, capture active carbonyl compounds, and hence inhibit the formation of AGEs (Song et al. 2021).

The anti‐glycation potential of ingredients derived from olive by‐products and olive leaves is gaining increasing attention. Troise et al. (2020) reported that the addition of 0.05%, 0.10%, and 0.20% spray‐dried olive mill wastewater significantly inhibited the formation of CML (with notable effects at 0.20%), CEL, and α‐dicarbonyl compound in cookies. Olive mill wastewater, a significant by‐product of olive oil, is a valuable source of polyphenols, including hydroxytyrosol, oleuropein, tyrosol, and verbascoside. Its phenolic content likely contributes to effective inhibition of early glycation stages and dicarbonyl compound formation (Navarro et al. 2015). In another study, olive leaf extract was found to reduce CEL levels by 42.16% and pentosidine levels by 37.30% at low concentrations of 0.01% and 0.05% (Navarro and Morales 2017). These anti‐glycation effects are largely attributed to its hydroxytyrosol content. However, as previously mentioned, similar effects were also observed with the addition of hydroxytyrosol alone; however, the reduction in CEL levels was less pronounced. It should be noted that the olive leaf extract was used at much lower concentrations and contained 100 times less hydroxytyrosol. This finding suggests that other phenolic compounds in the olive leaf extract may work synergistically with hydroxytyrosol to enhance its overall anti‐glycation activity.

As another plant extract, the addition of rooibos ( *Aspalathus linearis* ) extract to cookies at concentrations of 0.50% and 1.00% reduced CML and CEL levels by approximately 45.00%–70.00%, and MGO and GO levels by about 55.00%–75.00%, compared to control cookies. Furthermore, rooibos extract significantly decreased the levels of other AGEs, including MG‐H1, G‐H1, MOLD, GOLD, as well as dicarbonyl compounds such as 3‐DG, 3‐DGal, and glucosone (Chen, Lin, et al. 2022). Polyphenolic compounds such as orientin, isoorientin, vitexin, isovitexin, aspalathin, isoquercitrin, and rutin have been detected in rooibos‐enriched biscuits, and it has been reported that these compounds may reduce AGE levels by forming adducts with dicarbonyl compounds.

Studies have also examined the anti‐glycation effects of plant powders, whole plants, and isolated plant components in bakery products. Gao et al. (2020) reported that the addition of 1.00%–5.00% apple flower powder to cookies resulted in a dose‐dependent reduction of fluorescent AGEs by 44.77% and MGO by 60.36%. This study identified phlorizin as the primary bioactive compound contributing to the anti‐glycation activity of apple flower powder. In another study, adding 80.00 and 120.00 g of mashed spinach ( *Spinacia oleracea* ) microgreens directly to bread significantly decreased the fluorescent AGE levels in the crust, with reductions of 34.90% and 57.18%, respectively. Chlorophyll derivatives have been attributed to the effect of spinach microgreens in preventing AGE formation (Zhou et al. 2023). Huang et al. (2018) reporting that adding feruloylated oligosaccharides from maize bran to biscuits at concentrations of 1.25, 2.50, 5.00, 10.00, 20.00, and 40.00 mg/g flour resulted in dose‐dependent decreases in fluorescent AGE, 3‐DG, GO, and MGO levels. At a concentration of 20.00 mg/g flour, the highest inhibition was observed with a 90.00% reduction in fluorescent AGEs. Beyond this concentration, no further reduction in fluorescent AGEs was observed; however, higher doses resulted in a gradual decrease in dicarbonyl levels. It has been suggested that the feruloylated oligosaccharides in maize bran effectively inhibit AGE formation by reducing the levels of dicarbonyl compounds and limiting protein oxidation.

### 4.3 Common Types of AGEs and Analytical Techniques for Their Detection in Mitigation Studies Focused on Bakery Products

AGEs in foods are highly complex and varied, due to their derivation from a wide range of amino acids and carbonyl compounds. This complexity and variation contribute to the ongoing debate over the criteria for defining and detecting AGEs (Wei et al. 2018). The studies included in the systematic review predominantly detected fluorescent AGEs, with spectrofluorimetry being the only detection method used (Figures 3 and 4). In addition, five studies used only fluorescent AGEs as the sole marker to identify AGEs, without the support of other AGE markers or dicarbonyl compounds (Table 1). Certain AGEs, such as pentosidine, exhibit fluorescence intensity due to their chemical structures, whereas the majority of significant dietary AGEs found in foods, including CML, CEL, MG‐H1, and G‐H1, are non‐fluorescent (Zhang et al. 2020). Therefore, focusing only on fluorescent AGEs in strategies to reduce AGE formation in bakery products is insufficient, as it overlooks the significant presence of non‐fluorescent AGEs that dominate in these foods. A more effective approach would be to analyze different AGEs and their precursor dicarbonyl compounds to better understand and control AGE formation in bakery products. In this context, several studies have simultaneously examined multiple parameters, demonstrating concurrent reductions in fluorescent AGEs, CML levels, and dicarbonyl compounds such as MGO and GO (Table 1).

Among the commonly studied AGEs, CML is the most prominent and has been supported by CEL in some studies. Dicarbonyl compounds such as MGO and GO, which are precursors of these AGEs, have also been studied extensively (Figure 3). These findings are consistent with reports from other researchers who have identified CML and CEL as the most studied AGEs and MGO and GO as the most studied dicarbonyl compounds in food research (Hull et al. 2012; Maasen et al. 2021; Scheijen et al. 2016). There are also a few studies that have examined the levels of AGEs such as MG‐H1, G‐H1, MOLD, GOLD, and pentosidine as well as dicarbonyl compounds including 3‐DG, 3‐DGal, and glucosone (Figure 3). These compounds were usually detected by analytical techniques such as HPLC, UHPLC, and LC–MS/MS, and only two studies used the ELISA to determine CML levels (Figure 4). Various analytical techniques for AGE detection, including HPLC, GC–MS, spectrofluorimetry, LC–MS/MS, and UHPLC, offer distinct advantages in sensitivity, selectivity, and ease of use, but each method presents specific limitations such as derivatization requirements, environmental interferences, or high costs, necessitating careful consideration in method selection for accurate AGE quantification in food matrices (Wei et al. 2018). Comparative analyses have demonstrated that ELISA tends to overestimate CML concentrations in lipid‐rich foods relative to LC–MS/MS, leading to the recommendation against using immunochemical methods for accurate CML quantification in food matrices (Niquet‐Léridon et al. 2015). Current consensus favors LC–MS/MS and UHPLC as preferred methodologies for quantifying AGEs in food matrices, owing to their superior sensitivity and elimination of derivatization requirements (Zhang et al. 2020). In addition to these established methods, novel techniques for characterizing AGEs continue to be investigated, including molecularly imprinted polymers for selective detection and proteomic/peptidomic methods using mass spectrometry and bioinformatics (Zhang et al. 2020).

### 4.4 Strengths and Limitations

To our knowledge, this study is the first systematic review to provide a comprehensive overview of the literature evaluating the efficacy of various plant‐based ingredients and phytochemicals in reducing AGE formation in real bakery product matrices. The strength of this study lies in its systematic methodology that includes pre‐defined search criteria, specific keywords, databases, and strict inclusion/exclusion criteria. This approach minimizes bias, ensures reproducibility, and enhances the reliability of the findings compared to traditional reviews. Furthermore, the comprehensive quantitative presentation of reduction percentages for various AGEs and α‐dicarbonyl compounds in most of the included studies is another major strength of the review. However, potential limitations include the exclusion of non‐English articles and those for which the full text was not accessible. This could have led to a bias in language, thereby limiting the comprehensiveness and global relevance of the findings. Furthermore, the variation in experimental conditions among the included studies is another limitation. Direct comparison and generalization of the findings are challenging due to the variability in study designs, including differences in baking conditions (temperature and time), ingredient concentrations, and the analytical methods for AGE detection.

## 5 Conclusion

This systematic review highlights the potential activity of plant‐based ingredients and phytochemicals to reduce the formation of AGEs and α‐dicarbonyl compounds in bakery products. The examined studies demonstrated that various phytochemicals, including flavonoids, phenolic acids, stilbenes, and other polyphenols, as well as various plant extracts and by‐products, can effectively reduce the levels of specific AGEs and α‐dicarbonyl compounds in breads, cookies, and cakes. The phytochemicals most extensively studied for their anti‐glycation activity in bakery products include quercetin, naringenin, catechins, gallic acid, rutin, and ferulic acid. Additionally, grape and olive products are among the most researched plant‐based ingredients. The inhibition rate of various AGEs and α‐dicarbonyl compounds formed in bakery products by these plant‐based ingredients and phytochemicals ranges from < 10.00% to over 90.00%.

The anti‐glycation activity of polyphenolic compounds is primarily attributed to their chemical structures and antioxidant properties, which enable them to engage in various anti‐glycation mechanisms. Notably, the addition of plant extracts often exhibited greater anti‐glycation activity compared to the addition of individual phytochemicals, suggesting that synergistic interactions among diverse bioactive compounds may enhance their overall efficiency. However, the variability in baking conditions, product formulations, and analytical methods used for the determination of their concentrations across studies makes direct comparisons difficult. This underscores the need for standardized experimental protocols to improve the comparability and reproducibility of the findings (Chen, Li, et al. 2022).

Future research should focus on investigating dose–response relationships to identify the optimal concentrations of each compound for effective anti‐glycation. A comprehensive understanding of anti‐glycation activity in complex food systems will require the exploration of the extensive and largely unexplored spectrum of phytochemicals as well as the expansion of studies to include a wider range of AGEs and their precursors. In addition, it is crucial to evaluate the impact of these anti‐glycation strategies on the sensory and nutritional properties of bakery products to ensure consumer acceptance and maintain high food quality.

Evidence suggests that reducing dietary AGE intake may help mitigate disease risk (Dong, Li, et al. 2023) by enhancing metabolic risk factors (Sohouli et al. 2021), attenuating oxidative stress and inflammation (Kellow and Savige 2013), and promoting a healthier gut microbiome (Phuong‐Nguyen et al. 2023). In this context, the study is expected to provide valuable information for reformulating bakery products to reduce AGE content, which could improve public health by lowering dietary AGE intake.

## Author Contributions

**Busra Turan‐Demirci:** conceptualization (equal), data curation (equal), formal analysis (equal), funding acquisition (equal), investigation (equal), methodology (equal), visualization (equal), writing – original draft (equal). **Buket Gonen‐Colak:** conceptualization (equal), data curation (equal), formal analysis (equal), investigation (equal), visualization (equal), writing – review and editing (equal). **Zehra Buyuktuncer:** conceptualization (equal), formal analysis (equal), funding acquisition (equal), investigation (equal), supervision (equal), writing – review and editing (equal).

## Conflicts of Interest

The authors declare the following financial interests/personal relationships which may be considered as potential competing interests: The author reports that financial support was provided by the Scientific and Technological Research Council of Turkey. If there are other authors, they declare that they have no known competing financial interests or personal relationships that could have appeared to influence the work reported in this paper.

## Supporting information


Data S1.

Data S2.

Data S3.

## Data Availability

The data that support the findings of this study are available from the corresponding author upon reasonable request.
