# Supplementary material for: Exploring the Effects of Plant‐Based Ingredients and Phytochemicals on the Formation of Advanced Glycation End Products in Bakery Products: A Systematic Review
Source: Food Sci Nutr. 2025 Jun 30;13(7):e70534. doi: 10.1002/fsn3.70534 (PMC12208917; doi:10.1002/fsn3.70534)
Supplement: Supplementary file 2 — Data S2. [file FSN3-13-e70534-s001.docx]

| **KEY WORDS** | | |
| --- | --- | --- |
| “Advanced Glycation End Product*” | “Bakery Product*” | “Plant Extract*” |
| “Advanced Glycation Endproduct*” | “Baked Product*” | “Plant-Derived Compound*” |
| “Advanced Glycation End-Product*” | “Bakery Food*” | “Plant Derived Compound*” |
| “Advanced Glycosylation End Product*" | Pastr* | “Compounds, Plant-Derived” |
| “Advanced Glycosylation End-Product*" | “Pastry Product*” | “Plant-Derived Chemical*” |
| “Advanced Glycosylation Endproduct*" | Cookie* | “Plant Derived Chemical*” |
| “Glycosylation End Products, Advanced” | Biscuit* | “Chemicals, Plant-Derived” |
| “Glycosylation End Products” | Bread* | “Plant-Based Product*” |
| “Glycation End Products, Advanced” | Cake* | “Medicinal Plants*” |
| "Glycation End Products" | Muffin* | “Natural Compound*” |
| “Advanced Glycation” | Cracker* | “Natural Product*” |
| “Advanced Glycosylation” | Pasta* | “Natural Antioxidant*” |
| “AGEs” | Flour* | “Antioxidant*” |
| Carboxymethyl–lysine |  | “Natural Inhibitor*” |
| Carboxymethyllysine |  | “Bioactive Ingredient*” |
| N-epsilon-carboxymethyl-lysine |  | Phytochemical* |
| Carboxyethyl–lysine |  | Phytonutrient* |
| Carboxyethyllysine |  | Phenol* |
| N-epsilon-carboxymethyl-lysine |  | Polyphenol* |
| “Alpha-dicarbonyl Compound*” |  | “Phenolic Acid*” |
| Dicarbonyl* |  | Stilbene* |
| “Dicarbonyl Product*” |  | Curcumin* |
| Methylglyoxal |  | Lignan* |
| Methyl-glyoxal |  | Flavonoid* |
| Glyoxal |  | Flavanol* |
| “Amadori Product*” |  | Flavanone* |
| Pentosidine |  | Flavone* |
| Pyrraline |  | Flavonol* |
| Imidazolone |  | Isoflavon* |
| 3-Deoxyglucosone |  | Anthocyani* |
| “Fluorescent AGEs” |  | Terpen* |
| Maillard |  | Carotenoid* |
| “Maillard Reaction” |  | Xanthophyll* |
| “Maillard Reaction Product*” |  | Organosulfur* |
| "Non-Enzymatic Browning" |  | Saponin* |
|  |  | Phytoestrogen* |
|  |  | Phytosterol* |
|  |  | Alkaloid* |
|  |  | Glucosinolate* |
|  |  | Capsaicin* |
|  |  | Resveratrol |
