# Supplementary material for: Exploring the Effects of Plant‐Based Ingredients and Phytochemicals on the Formation of Advanced Glycation End Products in Bakery Products: A Systematic Review
Source: Food Sci Nutr. 2025 Jun 30;13(7):e70534. doi: 10.1002/fsn3.70534 (PMC12208917; doi:10.1002/fsn3.70534)
Supplement: Supplementary file 3 — Data S3. [file FSN3-13-e70534-s002.docx]

WEB of SCIENCE (n=408)

1

((((((((((((((((((((((((((((((((((TS=(“Advanced Glycation End Product*”)) OR TS=(“Advanced Glycation Endproduct*”)) OR TS=(“Advanced Glycation End-Product*”)) OR TS=(“Advanced Glycosylation End Product*")) OR TS=(“Advanced Glycosylation End-Product*")) OR TS=(“Advanced Glycosylation Endproduct*")) OR TS=(“Glycosylation End Products, Advanced”)) OR TS=(“Glycosylation End Products”)) OR TS=(“Glycation End Products, Advanced”)) OR TS=("Glycation End Products")) OR TS=(“Advanced Glycation”)) OR TS=(“Advanced Glycosylation”)) OR TS=(“AGEs”)) OR TS=(Carboxymethyl–lysine)) OR TS=(Carboxymethyllysine)) OR TS=(N-epsilon-carboxymethyl-lysine)) OR TS=(Carboxyethyl–lysine)) OR TS=(Carboxyethyllysine)) OR TS=(N-epsilon-carboxymethyl-lysine)) OR TS=(“Alpha-dicarbonyl Compound*”)) OR TS=(Dicarbonyl* )) OR TS=(“Dicarbonyl Product*”)) OR TS=(Methylglyoxal)) OR TS=(Methyl-glyoxal)) OR TS=(Glyoxal )) OR TS=(“Amadori Product*”)) OR TS=(Pentosidine)) OR TS=(Pyrraline)) OR TS=(Imidazolone)) OR TS=(3-Deoxyglucosone)) OR TS=(“Fluorescent AGEs”)) OR TS=(Maillard)) OR TS=(“Maillard Reaction”)) OR TS=(“Maillard Reaction Product*”)) OR TS=("Non-Enzymatic Browning")

2

((((((((((((TS=(“Bakery Product*”)) OR TS=(“Baked Product*”)) OR TS=(“Bakery Food*”)) OR TS=(Pastr*)) OR TS=(“Pastry Product*”)) OR TS=(Cookie*)) OR TS=(Biscuit*)) OR TS=(Bread*)) OR TS=(Cake*)) OR TS=(Muffin*)) OR TS=(Cracker*)) OR TS=(Pasta* )) OR TS=(Flour*)

3

((((((((((((((((((((((((((((((((((((((((TS=(“Plant Extract*”)) OR TS=(“Plant-Derived Compound*”)) OR TS=(“Plant Derived Compound*”)) OR TS=(“Compounds, Plant-Derived”)) OR TS=(“Plant-Derived Chemical*”)) OR TS=(“Plant Derived Chemical*”)) OR TS=(“Chemicals, Plant-Derived”)) OR TS=(“Plant-Based Product*”)) OR TS=(“Medicinal Plants*”)) OR TS=(“Natural Compound*”)) OR TS=(“Natural Product*”)) OR TS=(“Natural Antioxidant*”)) OR TS=(“Antioxidant*”)) OR TS=(“Natural Inhibitor*”)) OR TS=(“Bioactive Ingredient*”)) OR TS=(Phytochemical*)) OR TS=(Phytonutrient*)) OR TS=(Phenol*)) OR TS=(Polyphenol*)) OR TS=(“Phenolic Acid*”)) OR TS=(Stilbene*)) OR TS=(Curcumin*)) OR TS=(Lignan*)) OR TS=(Flavonoid*)) OR TS=(Flavanol*)) OR TS=(Flavanone*)) OR TS=(Flavone*)) OR TS=(Flavonol*)) OR TS=(Isoflavon*)) OR TS=(Anthocyani*)) OR TS=(Terpen*)) OR TS=(Carotenoid*)) OR TS=(Xanthophyll*)) OR TS=(Organosulfur*)) OR TS=(Saponin*)) OR TS=(Phytoestrogen*)) OR TS=(Phytosterol*)) OR TS=(Alkaloid*)) OR TS=(Glucosinolate*)) OR TS=(Capsaicin*)) OR TS=(Resveratrol)

#1 AND #2 AND #3

PUBMED (n=130)

1

(((((((((((((((((((((((((((((((((("Advanced Glycation End Product*"[Title/Abstract]) OR ("Advanced Glycation Endproduct*"[Title/Abstract])) OR ("Advanced Glycation End-Product*"[Title/Abstract])) OR ("Advanced Glycosylation End Product*"[Title/Abstract])) OR ("Advanced Glycosylation End-Product*"[Title/Abstract])) OR ("Advanced Glycosylation Endproduct*"[Title/Abstract])) OR ("Glycosylation End Products, Advanced"[Title/Abstract])) OR ("Glycosylation End Products"[Title/Abstract])) OR ("Glycation End Products, Advanced"[Title/Abstract])) OR ("Glycation End Products"[Title/Abstract])) OR ("Advanced Glycation"[Title/Abstract])) OR ("Advanced Glycosylation"[Title/Abstract])) OR ("AGEs"[Title/Abstract])) OR (Carboxymethyl–lysine[Title/Abstract])) OR (Carboxymethyllysine[Title/Abstract])) OR (N-epsilon-carboxymethyl-lysine[Title/Abstract])) OR (Carboxyethyl–lysine[Title/Abstract])) OR (Carboxyethyllysine[Title/Abstract])) OR (N-epsilon-carboxymethyl-lysine[Title/Abstract])) OR ("Alpha-dicarbonyl Compound*"[Title/Abstract])) OR (Dicarbonyl*[Title/Abstract])) OR ("Dicarbonyl Product*"[Title/Abstract])) OR (Methylglyoxal[Title/Abstract])) OR (Methyl-glyoxal[Title/Abstract])) OR (Glyoxal[Title/Abstract])) OR ("Amadori Product*"[Title/Abstract])) OR (Pentosidine[Title/Abstract])) OR (Pyrraline[Title/Abstract])) OR (Imidazolone[Title/Abstract])) OR (3-Deoxyglucosone[Title/Abstract])) OR ("Fluorescent AGEs"[Title/Abstract])) OR (Maillard[Title/Abstract])) OR ("Maillard Reaction"[Title/Abstract])) OR ("Maillard Reaction Product*"[Title/Abstract])) OR ("Non-Enzymatic Browning"[Title/Abstract])

2

(((((((((((("Bakery Product*"[Title/Abstract]) OR ("Baked Product*"[Title/Abstract])) OR ("Bakery Food*"[Title/Abstract])) OR (Pastr*[Title/Abstract])) OR ("Pastry Product*"[Title/Abstract])) OR (Cookie*[Title/Abstract])) OR (Biscuit*[Title/Abstract])) OR (Bread*[Title/Abstract])) OR (Cake*[Title/Abstract])) OR (Muffin*[Title/Abstract])) OR (Cracker*[Title/Abstract])) OR (Pasta*[Title/Abstract])) OR (Flour*[Title/Abstract])

3

(((((((((((((((((((((((((((((((((((((((("Plant Extract*"[Title/Abstract]) OR ("Plant-Derived Compound*"[Title/Abstract])) OR ("Plant Derived Compound*"[Title/Abstract])) OR ("Compounds, Plant-Derived"[Title/Abstract])) OR ("Plant-Derived Chemical*"[Title/Abstract])) OR ("Plant Derived Chemical*"[Title/Abstract])) OR ("Chemicals, Plant-Derived"[Title/Abstract])) OR ("Plant-Based Product*"[Title/Abstract])) OR ("Medicinal Plants*"[Title/Abstract])) OR ("Natural Compound*"[Title/Abstract])) OR ("Natural Product*"[Title/Abstract])) OR ("Natural Antioxidant*"[Title/Abstract])) OR ("Antioxidant*"[Title/Abstract])) OR ("Natural Inhibitor*"[Title/Abstract])) OR ("Bioactive Ingredient*"[Title/Abstract])) OR (Phytochemical*[Title/Abstract])) OR (Phytonutrient*[Title/Abstract])) OR (Phenol*[Title/Abstract])) OR (Polyphenol*[Title/Abstract])) OR ("Phenolic Acid*"[Title/Abstract])) OR (Stilbene*[Title/Abstract])) OR (Curcumin*[Title/Abstract])) OR (Lignan*[Title/Abstract])) OR (Flavonoid*[Title/Abstract])) OR (Flavanol*[Title/Abstract])) OR (Flavanone*[Title/Abstract])) OR (Flavone*[Title/Abstract])) OR (Flavonol*[Title/Abstract])) OR (Isoflavon*[Title/Abstract])) OR (Anthocyani*[Title/Abstract])) OR (Terpen*[Title/Abstract])) OR (Carotenoid*[Title/Abstract])) OR (Xanthophyll*[Title/Abstract])) OR (Organosulfur*[Title/Abstract])) OR (Saponin*[Title/Abstract])) OR (Phytoestrogen*[Title/Abstract])) OR (Phytosterol*[Title/Abstract])) OR (Alkaloid*[Title/Abstract])) OR (Glucosinolate*[Title/Abstract])) OR (Capsaicin*[Title/Abstract])) OR (Resveratrol[Title/Abstract])

#1 AND #2 AND #3

SCOPUS (n=689)

TITLE-ABS-KEY ( "Advanced Glycation End Product*" OR "Advanced Glycation Endproduct*" OR "Advanced Glycation End-Product*" OR "Advanced Glycosylation End Product*" OR "Advanced Glycosylation End-Product*" OR "Advanced Glycosylation Endproduct*" OR "Glycosylation End Products, Advanced" OR "Glycosylation End Products" OR "Glycation End Products, Advanced" OR "Glycation End Products" OR "Advanced Glycation" OR "Advanced Glycosylation" OR "AGEs" OR carboxymethyl--lysine OR carboxymethyllysine OR n-epsilon-carboxymethyl-lysine OR carboxyethyl--lysine OR carboxyethyllysine OR n-epsilon-carboxymethyl-lysine OR "Alpha-dicarbonyl Compound*" OR dicarbonyl* OR "Dicarbonyl Product*" OR methylglyoxal OR methyl-glyoxal OR glyoxal OR "Amadori Product*" OR pentosidine OR pyrraline OR imidazolone OR 3-deoxyglucosone OR "Fluorescent AGEs" OR maillard OR "Maillard Reaction" OR "Maillard Reaction Product*" OR "Non-Enzymatic Browning" ) AND TITLE-ABS-KEY ( "Bakery Product*" OR "Baked Product*" OR "Bakery Food*" OR pastr* OR "Pastry Product*" OR cookie* OR biscuit* OR bread* OR cake* OR muffin* OR cracker* OR pasta* OR flour* ) AND TITLE-ABS-KEY ( "Plant Extract*" OR "Plant-Derived Compound*" OR "Plant Derived Compound*" OR "Compounds, Plant-Derived" OR "Plant-Derived Chemical*" OR "Plant Derived Chemical*" OR "Chemicals, Plant-Derived" OR "Plant-Based Product*" OR "Medicinal Plants*" OR "Natural Compound*" OR "Natural Product*" OR "Natural Antioxidant*" OR "Antioxidant*" OR "Natural Inhibitor*" OR "Bioactive Ingredient*" OR phytochemical* OR phytonutrient* OR phenol* OR polyphenol* OR "Phenolic Acid*" OR stilbene* OR curcumin* OR lignan* OR flavonoid* OR flavanol* OR flavanone* OR flavone* OR flavonol* OR isoflavon* OR anthocyani* OR terpen* OR carotenoid* OR xanthophyll* OR organosulfur* OR saponin* OR phytoestrogen* OR phytosterol* OR alkaloid* OR glucosinolate* OR capsaicin* OR resveratrol )
